# Ume6 Acts as a Stable Platform To Coordinate Repression and Activation of Early Meiosis-Specific Genes in Saccharomyces cerevisiae

**DOI:** 10.1128/MCB.00378-20

**Published:** 2021-06-23

**Authors:** Sheetal A. Raithatha, Shivani Vaza, M. Touhidul Islam, Brianna Greenwood, David T. Stuart

**Affiliations:** a Department of Biochemistry, University of Alberta, Edmonton, Alberta, Canada

**Keywords:** Gcn5, Ime1, Rpd3, Ume6, meiosis, sporulation, transcription factors, yeasts

## Abstract

In response to nutrient starvation, the budding yeast Saccharomyces cerevisiae abandons mitotic proliferation and embarks on a differentiation process that leads through meiosis to the formation of haploid spores. This process is driven by cascading waves of meiosis-specific-gene expression. The early meiosis-specific genes are repressed during mitotic proliferation by the DNA-binding protein Ume6 in combination with repressors Rpd3 and Sin3. The expression of meiosis-specific transcription factor Ime1 leads to activation of the early meiosis-specific genes. We investigated the stability and promoter occupancy of Ume6 in sporulating cells and determined that it remains bound to early meiosis-specific gene promoters when those genes are activated. Furthermore, we find that the repressor Rpd3 remains associated with Ume6 after the transactivator Ime1 has joined the complex and that the Gcn5 and Tra1 components of the SAGA complex bind to the promoter of *IME2* in an Ime1-dependent fashion to induce transcription of the early meiosis-specific genes. Our investigation supports a model whereby Ume6 provides a platform allowing recruitment of both activating and repressing factors to coordinate the expression of the early meiosis-specific genes in Saccharomyces cerevisiae.

## INTRODUCTION

Modulation of gene expression is crucial to cellular development and differentiation processes where large sets of genes must be activated and deactivated in a tightly orchestrated fashion to achieve changes in cell phenotype and morphology. Coordinated assembly, modification, and disassembly of transcription factor complexes are major mechanisms that can be employed to control regulated transcription (1). Gamete formation (sporulation) in Saccharomyces cerevisiae is a well-studied developmental process that is driven by temporally regulated waves of gene expression (2). Upon nitrogen and glucose starvation, S. cerevisiae abandons mitotic proliferation and initiates a differentiation program that leads through meiosis to the formation of haploid spores. The combination of nutrient signaling and *MAT***a**/*MAT*α genome status triggers a cascade of gene expression that activates previously silent genes and promotes progression through premeiotic DNA replication, elevated rates of homologous recombination, reductional and equational chromosome division, and finally, spore morphogenesis (3–5). An immediate response to starvation is induction of the Ime1 transcription factor, which has been referred to as the “master regulator” of meiosis and the sporulation process (6). *IME1* is strictly required for the induction of early meiosis-specific-gene expression (7–9).

The early gene family consists of genes whose products function to promote and regulate early events of the sporulation program, including premeiotic DNA replication, homologous recombination, and progression into the meiotic chromosome divisions (3, 5). Most of these genes are held silent during mitotic proliferation. A major component of the regulation of these genes is imposed through the conserved URS1 DNA sequence found upstream from the open reading frames of all members of this gene family (10). The URS1 DNA sequence is a binding site for the Ume6 protein (11). During mitotic proliferation, Ume6 bound to URS1 recruits histone deacetylase Rpd3, corepressor Sin3, and chromatin remodeling factor Isw2 (12, 13). These factors repress early meiosis-specific-gene expression in part through deacetylation of the surrounding histones and formation of inaccessible chromatin structures (14–16). Mutational inactivation of Ume6, Rpd3, or Sin3 results in derepression of early meiosis-specific genes, allowing their expression during vegetative growth (17–19). These observations led to the proposal that Ume6 acts as a repressive factor to control early meiosis-specific genes. Induction of the early meiosis-specific genes is dependent upon Ime1 expression and nuclear localization (7, 20). Ime1 has a transcriptional activation domain and transcriptional activation capabilities when tethered to DNA but displays no intrinsic sequence-specific DNA binding capability (9, 21). Ime1 is not associated with early meiosis-specific gene promoters in *ume6* mutant strains, suggesting that Ume6 recruits the Ime1 *trans* activator to trigger early gene expression during sporulation (22, 23). Two-hybrid experiments revealed that Ime1 can bind Ume6 and that mutations in threonine 99 of Ume6 block this interaction (22). Additionally, Ime1 mutants that can bind to Ume6 but lack the sequences encoding the transcription-activating domain are defective in inducing meiosis-specific-gene expression (21). The observation that a LexA-Ume6 fusion protein confers Ime1-dependent activation on a LexA operator-regulated reporter gene is consistent with the proposal that Ume6 represses meiosis-specific genes through recruitment of Sin3-Rpd3 repressors and that the complex is converted into an activator upon binding Ime1 (22, 24). This parsimonious model is further supported by the observation that a Gal4 activation domain (GAD)-Ume6 fusion protein allows meiosis in cells lacking Ime1 (24).

A competing model for regulation of early meiosis-specific genes was proposed by Mallory et al., who showed that Ume6 was degraded in an Ime1-dependent fashion, removing the repressive complex from early gene promoters (25). It was further proposed that Ume6 destruction and early meiosis-specific-gene expression was dependent upon the Cdc20-activating subunit of the anaphase promoting complex (APC^Cdc20^) (25). This model provided no mechanism for meiosis-specific induction of genes beyond the derepressed level achieved by loss of Ume6.

Cdc20 performs essential functions during mitotic proliferation and during progress through meiosis (26). During the sporulation process, Cdc20 is expressed in the middle phase of meiosis, and cells subjected to meiosis-specific depletion of Cdc20 arrest at metaphase I, displaying metaphase I spindles, complete DNA replication, and high levels of Pds1 (26, 27). Proteins encoded by early genes *REC8* and *HOP1* and middle genes *CLB5*, *NDT80*, and *CLB1* accumulate with normal kinetics in sporulating cells depleted of Cdc20 (28). Thus, it is surprising that Cdc20 should be implicated in triggering the expression of the early meiosis-specific genes. The APC subunit Cdh1 is expressed during sporulation but is not critical for progression through sporulation and spore formation (27, 29, 30). In contrast, Ama1 is a meiosis-specific APC activating subunit (31). Cells lacking Ama1 progress into the early phase of the sporulation program with wild-type kinetics (27, 32, 33), although some strain backgrounds display defects in chromosome divisions and spore wall defects (34, 35). In contrast to the lack of a demonstrable requirement for APC activity to allow progression into and through the early phase of sporulation, protein degradation by autophagy is essential for sporulation (36, 37). Cells deficient in autophagy factor Atg12 arrest prior to meiotic DNA replication and lose viability (37). Ume6 is not a substrate for degradation by the autophagy process in S. cerevisiae. Indeed, Ume6 is a regulator of the gene encoding the essential autophagy factor Atg8 (36).

The lysine acetyltransferase Gcn5 is a component of the SAGA and SLIK complexes, which interact with transcriptional activators to promote active transcription (38, 39). Gcn5 is also required for the induction of meiosis-specific genes and for sporulation (40). However, previous studies have not demonstrated that Gcn5 or SAGA specifically associates with meiosis-specific transcriptional activators. Histones surrounding the URS1 sites in the *IME2* promoter display a Gcn5-dependent increase in acetylation, suggesting that SAGA may be recruited to this promoter, but it has also been proposed that Gcn5 acetylates Ume6 to promote Ume6’s destruction (41).

Activation of the early meiosis-specific genes by destruction of Ume6 is inconsistent with a requirement for Ume6 to recruit Ime1 in the role of a *trans* activator. Additionally, the failure of *ume6* deletion strains to effectively induce early meiosis-specific genes and progress through the sporulation program is inconsistent with a purely repressive role for Ume6 (42). Our investigation of early meiosis-specific-gene regulation suggests that Ume6 remains bound to the promoters of early meiosis-specific genes *IME2* and *SPO13* throughout the early stages of the sporulation program, where it creates a platform for the recruitment of both transcriptional repressor Rpd3 and activator Ime1.

## RESULTS

### Ume6 is stable during the early phase of the sporulation program.

**FIG 1:**
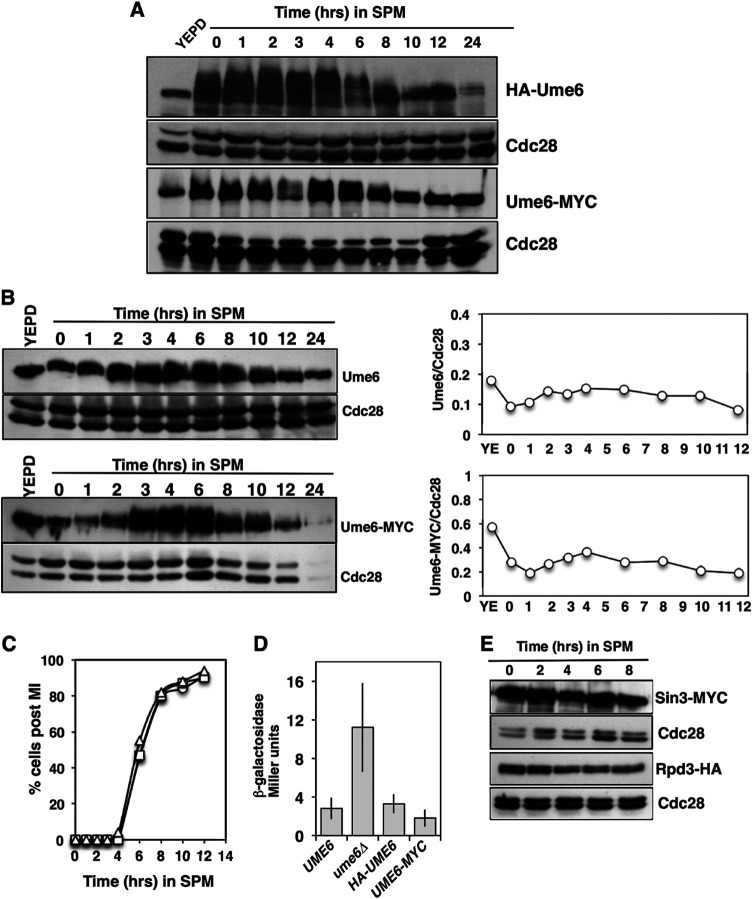
Ume6 protein abundance does not decrease during the early phase of sporulation. (A) Western blots detecting the abundance of HA-Ume6 (top) and Ume6-MYC (bottom) during vegetative growth (YEPD) or following induction of the sporulation program in sporulation medium (SPM). Cdc28 abundance was monitored as a control for protein loading. Ume6 and Cdc28 were probed on the same membranes. (B) The signals from untagged Ume6 probed with an anti-Ume6 antibody, Ume6-MYC probed with anti-MYC antibody, and loading control Cdc28 were quantified and are presented as the Ume6/Cdc28 ratios. (C) Percentages of diploid *UME6*/*UME6* (circles), *HA-UME6*/*HA-UME6* (squares), and *UME6-MYC*/*UME6-MYC* (triangles) cells displaying two or more segregated chromatin masses following inoculation into SPM. (D) β-Galactosidase activity produced in actively proliferating cells harboring a centromeric *IME2*-LacZ reporter plasmid. Activity is reported in Miller units. Data are reported as the mean levels of activity measured from three independent transformants. The error bars reflect standard deviations. (E) Western blot analysis of Sin3-MYC, Rpd3-HA, and Cdc28 abundance in cultures induced to initiate synchronous sporulation by inoculation into SPM. Sin3-MYC samples were separated on a 6% SDS–polyacrylamide gel, and duplicate samples were separated on a 10% gel to probe for Cdc28. Rpd3-HA samples were electrophoresed on duplicate 10% SDS–polyacrylamide gels; one was probed for Rpd3-HA and a duplicate probed for Cdc28.

We monitored the abundance of hemagglutinin-tagged Ume6 (HA-Ume6) in a population of cells induced to synchronously initiate sporulation. Protein extracts prepared under denaturing conditions and analyzed by Western blotting revealed that the HA-Ume6 abundance did not display a significant reduction during the early phase of sporulation from 0 to 8 h (Fig. 1A). Ume6 did display slow mobility species as the cells initiated the sporulation program (0 to 6 h). This reduced mobility has been observed by others and was largely attributed to phosphorylation of Ume6 by kinases Rim11 and Rim15 (43). Some reduction in the Ume6 abundance was noted in the later phases of sporulation; however, during the time period in which early genes are activated (1 to 4 h), there did not appear to be a dramatic reduction in the Ume6 abundance. The HA-tagged version of Ume6 was functional in that *HA-UME6* cells displayed sporulation timing and frequency similar to those of *UME6* cells (Fig. 1C). Additionally, *HA-UME6* was able to repress an *IME2*-LacZ reporter gene in actively proliferating cells to an extent similar to that observed with *UME6* (Fig. 1D). A second version of Ume6, with a carboxyl-terminal MYC tag, was also generated. In diploid cells induced to initiate the sporulation program, Ume6-MYC displayed behavior similar to that of HA-Ume6 (Fig. 1A). The abundance of Ume6-MYC was not reduced during the early phase of sporulation, but Ume6-MYC did display more slowly migrating species that were not apparent during the later phases of sporulation (Fig. 1A). Like HA-Ume6, Ume6-MYC was functional, based upon the ability of *UME6-MYC-*expressing cells to undergo meiosis and sporulation with timing and frequency similar to those of a *UME6* strain and to enforce repression of an *IME2*-LacZ reporter gene during mitotic proliferation (Fig. 1C and D). Although the added HA or MYC epitope tags did not appear to alter Ume6 function with regard to progression through the sporulation program, we considered that the appended epitope tag sequences may have influenced Ume6’s stability during this process. To test this possibility, protein extracts were prepared from a strain expressing the native untagged Ume6 (DSY1089) and subjected to Western blot analysis using an anti-Ume6 antibody directed against residues 339 to 808. This analysis revealed that the native Ume6 protein displayed a pattern of abundance similar to those displayed by the epitope-tagged versions of Ume6 (Fig. 1B). A Li-COR Odyssey scanner was used to quantify the Western blot signals for this experiment, and the Ume6/Cdc28 ratios were plotted (Fig. 1B). This analysis revealed that the Ume6 abundance was higher in cells proliferating in yeast extract-peptone-dextrose (YEPD) medium and that there was some fluctuation in Ume6 abundance throughout the sporulation program but no dramatic reduction during the early and middle periods for sporulation (2 to 8 h), when early meiosis-specific genes are induced. A similar result was observed when monitoring Ume6-MYC (Fig. 1B, bottom).

**FIG 2:**
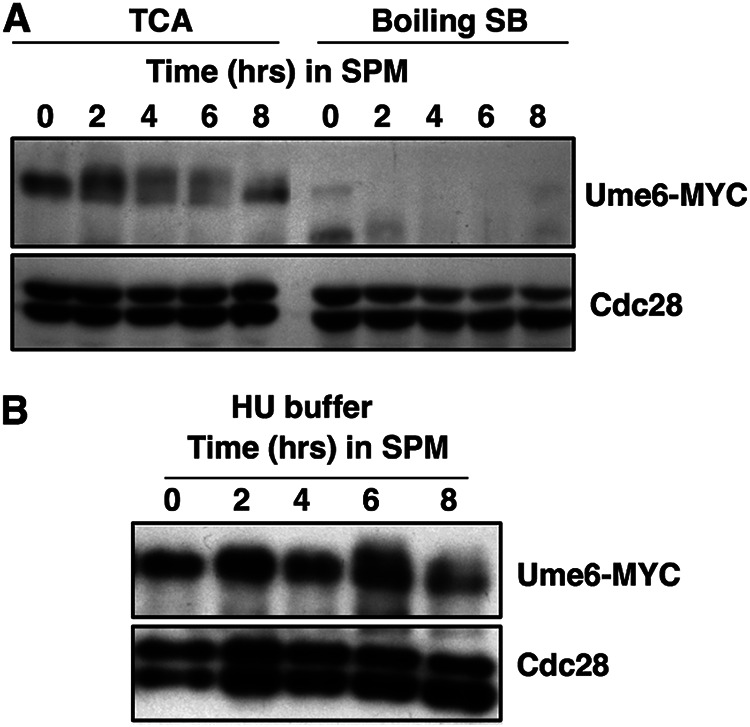
Denaturing extraction with TCA improves the detection of Ume6-MYC over extraction by boiling in SDS sample buffer. Samples of *UME6-MYC*/*UME6-MYC* cells were collected at the indicated time points following the induction of sporulation. (A) Protein extracts were prepared by either vortexing cell pellets with glass beads in 20% trichloroacetic acid (TCA) or by boiling in 1× SDS sample buffer (SB). (B) Cells harvested at the indicated time points following induction of sporulation were lysed with NaOH and the proteins precipitated by addition of TCA prior to resuspending in 8 M urea–5% SDS. Samples were probed by Western blotting for Ume6-MYC and Cdc28 as indicated.

As these findings differ from those reported previously, we investigated the abundance of Ume6 that could be detected when the protein extracts were prepared by boiling cell pellets in SDS sample buffer (25, 44). When protein extracts were prepared by vortexing with glass beads in trichloroacetic acid (TCA) followed by precipitation, as described in Materials and Methods, Ume6-MYC could be detected during the 2 to 8 h following induction of the sporulation program (Fig. 2A, TCA). In contrast, when protein samples were prepared by boiling cell pellets in SDS sample buffer, the signal for Ume6-MYC was specifically reduced (Fig. 2A, Boiling SB). A similar reduction in the Ume6 signal was observed when the protein extracts were prepared by mechanical lysis under nondenaturing conditions (data not shown). Knop et al. have described a method to lyse cells in NaOH, followed by TCA precipitation and solubilization in buffers containing urea and SDS (45). This method has been reported to efficiently extract proteins from sporulating cells. A much lower reduction in the Ume6-MYC signal was observed when extracts were prepared with this method than from boiling cells in SDS sample buffer (Fig. 2B).

These observations suggest that, *in vivo*, Ume6 is not dramatically degraded during the early phase of the sporulation program but that it may be labile upon cell lysis or difficult to extract from sporulating cells. The relative stability of Ume6 that we observed is consistent with earlier findings that Ume6 not only performs an important role for imposing repression on early meiosis-specific genes during vegetative growth but is also required for the effective induction of this gene family during sporulation.

### Repressors Sin3 and Rpd3 are stable throughout the sporulation process.

Ume6 imposes repression on meiosis-specific genes by forming a complex with histone deacetylase Rpd3 and corepressor Sin3. To determine whether or not the two repressive factors were degraded upon entry into and progression through sporulation, we monitored the abundance of Sin3-MYC and Rpd3-HA during the course of a synchronous sporulation (Fig. 1E). Sin3-MYC was present in actively proliferating cells (*T* = 0) and could also be detected during the early portion of the sporulation time course, up to 8 h, when the early meiosis-specific genes are induced. During the early phase of sporulation, the abundance of Sin3-MYC remained relatively constant (Fig. 1E, time points 0 to 8 h). Similarly, Rpd3-HA could be detected as the cells progressed through the sporulation process (Fig. 1E). There was little reduction in Sin3-MYC or Rpd3-HA through the 2- to 6-h time points, when the early meiosis-specific genes are induced (Fig. 1E).

### Cdc20-associated APC activity is not required for induction of the early meiosis-specific genes.

**FIG 3:**
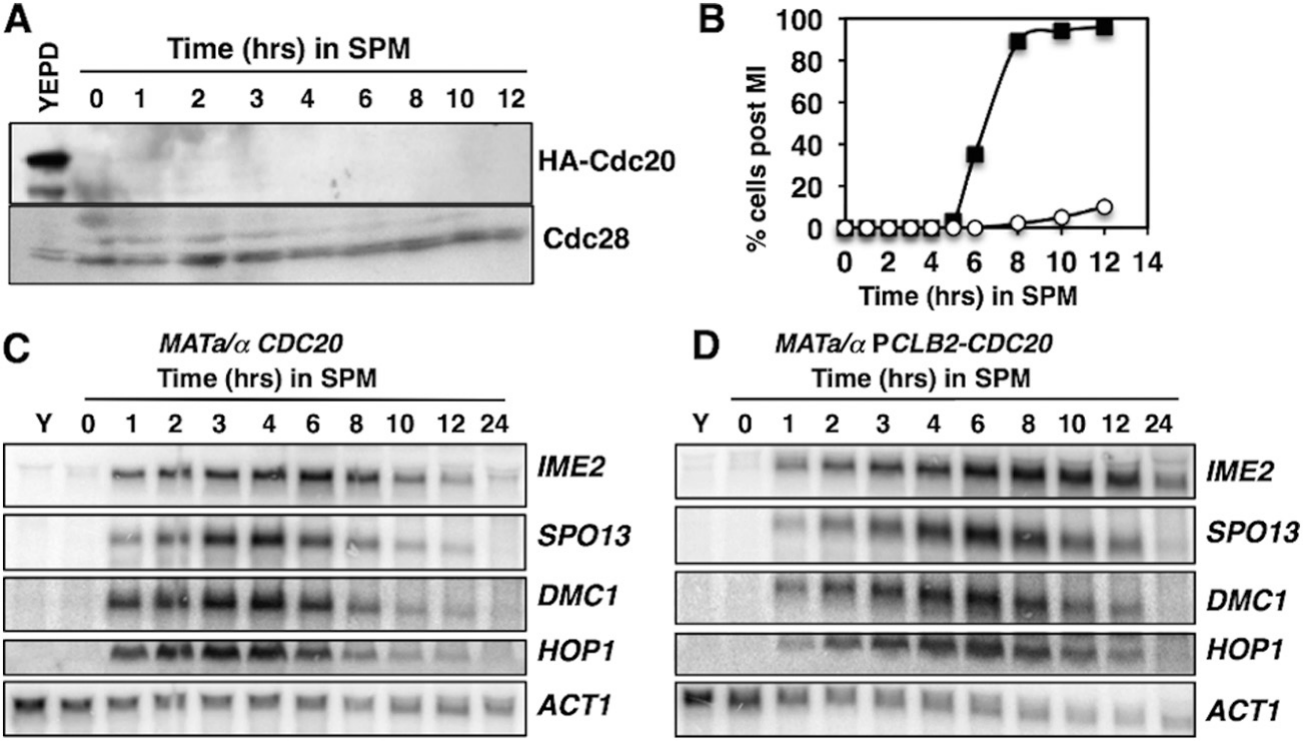
Cdc20 is not required for the induction of early meiosis-specific genes. (A) Western blot analysis of HA-Cdc20 in actively proliferating P*CLB2*-*CDC20*/P*CLB2*-*CDC20* cells (YEPD) and in cells induced to initiate sporulation by inoculation into SPM. The upper half of the membrane was probed with an anti-HA antibody, and the lower portion of the membrane was probed for Cdc28 as a loading control. (B) Percentages of diploid *CDC20*/*CDC20* (squares) and P*CLB2*-*CDC20*/P*CLB2*-*CDC20* (circles) cells displaying two or more segregated DAPI-stained chromatin masses. (C, D) Northern blot analysis of transcripts of early meiosis-specific genes *IME2*, *SPO13*, *DMC1*, and *HOP1*. The *ACT1* transcript was included as a control. RNA was collected from actively proliferating cultures (Y) or at the indicated time points following induction of sporulation.

It has been proposed that APC^Cdc20^ regulates early meiosis-specific-gene expression through control of Ume6’s stability (25). Although our data suggest that the abundance of Ume6 does not decrease significantly in the early phases of sporulation, we investigated whether APC^Cdc20^ had a role in regulating early meiosis-specific-gene expression. *CDC20* is an essential gene, so to test its effects on Ume6’s stability and early meiosis-specific-gene expression, we made use of a *CDC20* allele in which the open reading frame is placed under the regulation of the *CLB2* promoter, which is expressed exclusively during mitotic proliferation (26). These cells effectively expressed Cdc20 during mitotic proliferation (Fig. 3A, YEPD), but upon completion of the mitotic M phase and entry into the sporulation program, Cdc20’s abundance was reduced and undetectable when cells were transferred into sporulation medium (Fig. 3A, 0 to 12 h) (26). This strain is reported to display premeiotic DNA replication with normal timing and formation of a metaphase spindle (27). However, the strain undergoes arrest in meiosis I (MI) (Fig. 3B) (26). Although the P*CLB2*-*CDC20* strain displayed arrest in MI, we could observe no significant difference in the timing of induction or the abundance of transcripts of early genes *IME2*, *SPO13*, *DMC1*, and *HOP1* (Fig. 3C and D). The abundance of the early gene transcripts declined as *CDC20*/*CDC20* cells progressed through meiosis I (MI) and meiosis II (MII) (Fig. 3C). In contrast, the early gene transcripts persisted for a longer time in the P*CLB2-CDC20*/P*CLB2-CDC20* strain (Fig. 3D), consistent with arrest of the strain at metaphase I (27). These observations are inconsistent with a scenario where Cdc20-dependent destruction of Ume6 is required to release these genes from repression.

### Ume6 remains bound to early gene promoters throughout the early phase of sporulation.

**FIG 4:**
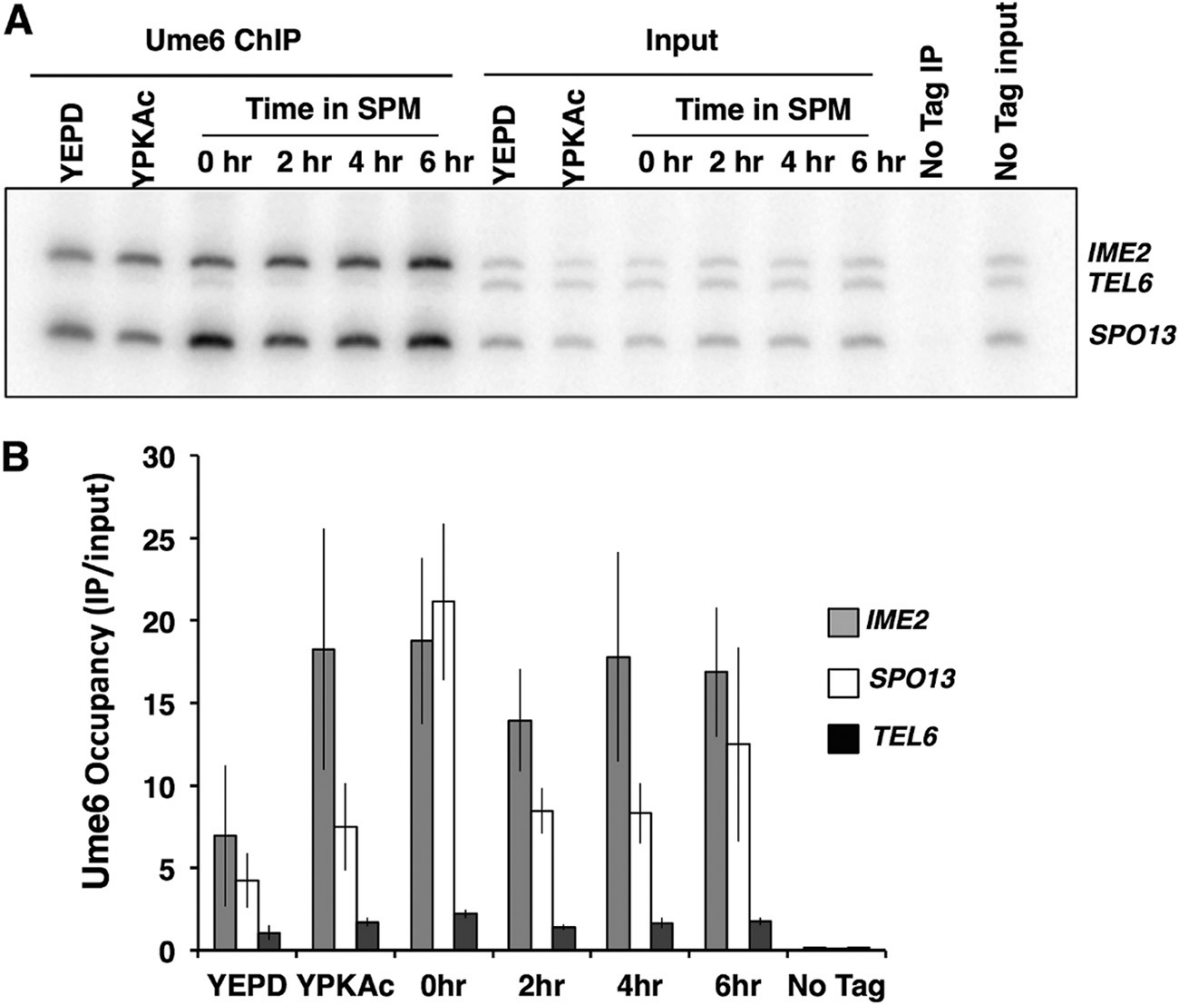
Ume6 remains bound to early meiosis-specific gene promoters during sporulation. (A) PCR analysis of Ume6-MYC immunoprecipitates (Ume6 ChIP) or samples of the input extracts (Input) collected from cultures growing in medium supplemented with glucose (YEPD) or acetate (YPKAc) or from cells at the indicated times following inoculation into sporulation medium (SPM). The “No Tag IP” label is used for immunoprecipitate or input DNA from an untagged *UME6* strain. (B) Quantitative analysis of the PCR products from Ume6-MYC immunoprecipitates. Data presented are mean IP/input ratios from three repetitions; error bars reflect standard deviations.

Although we observed that the Ume6 abundance was not reduced during the early phase of the sporulation program, we considered the possibility that DNA-bound Ume6 is specifically targeted or that Ume6 is displaced from early gene promoters. We investigated the occupancy of Ume6 at the promoters of the early meiosis-specific genes *SPO13* and *IME2*, as well as the telomere of chromosome VI, which has no identifiable URS1 binding site for Ume6 (46). Chromatin immunoprecipitation (ChIP) was performed to investigate the abundance of Ume6 present on these early meiosis-specific gene promoters. PCR analysis of Ume6-MYC immunoprecipitates from mitotically proliferating cells (YEPD medium), as well as cells in the early phase of sporulation (0 to 6 h), displayed enrichment for products amplified from the *SPO13* and *IME2* promoters relative to the *TELVI* signal, indicating occupancy of Ume6 on the early gene promoters. The *SPO13* and *IME2* product signals did not display any significant reduction during the early phase of the sporulation program, when *SPO13* and *IME2* mRNA accumulated maximally (Fig. 4). These observations are consistent with the proposal that Ume6 remains associated with early meiosis-specific gene promoters both when these genes are repressed and once they become activated.

### Chromatin-bound Ume6 binds both activators and repressors of meiosis-specific transcription.

**FIG 5:**
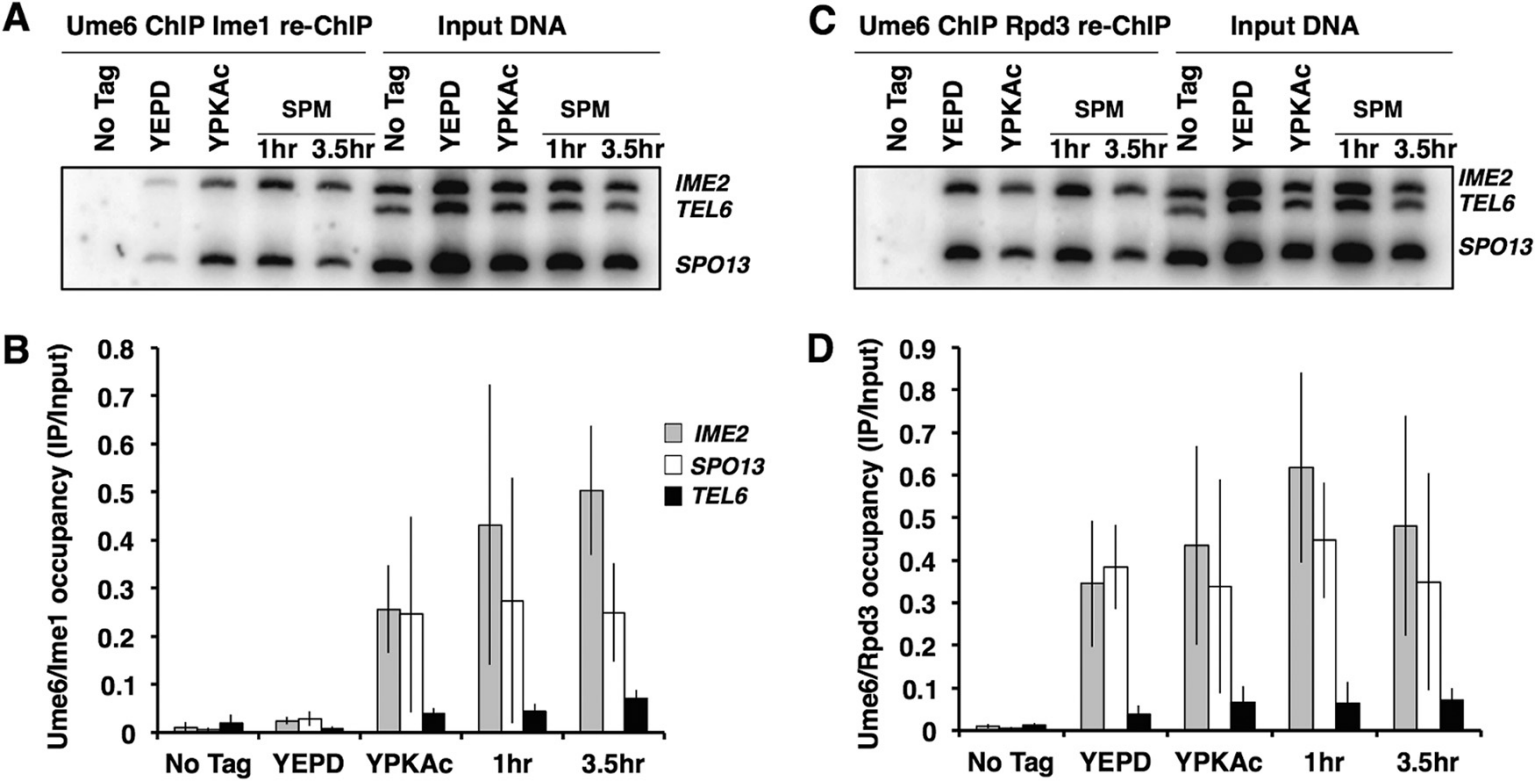
Ime1 and Rpd3 are bound to Ume6 during sporulation. (A) PCR analysis of Ume6-MYC immunoprecipitates that were reprecipitated with anti-HA antibodies to isolate Ime1-HA. Immunoprecipitate from a *UME6*-MYC *IME1* strain (No tag) or *UME6*-MYC *IME1*-HA strain actively proliferating in medium supplemented with glucose (YEPD) or acetate (YPKAc) or induced to sporulate (SPM). (B) Quantitative analysis of the PCR products from Ume6-MYC Ime1-HA immunoprecipitates. (C) PCR analysis of Ume6-MYC immunoprecipitates that were reprecipitated with anti-HA antibodies to isolate Rpd3-HA. Immunoprecipitate from a *UME6*-MYC *RPD3* strain (No tag) or *UME6*-MYC *RPD3*-HA strain actively proliferating in medium supplemented with glucose (YEPD) or acetate (YPKAc) or induced to sporulate (SPM). (D) Quantitative analysis of the PCR products from Ume6-MYC Rpd3-HA immunoprecipitates. Data presented are mean IP/input ratios from three repetitions; error bars reflect standard deviations.

Expression of the Ime1 transcription factor is required for induction of the early meiosis-specific genes (7, 21). Ime1 displays no specific DNA binding activity of its own but can physically interact with Ume6 in a yeast two-hybrid assay, leading to the suggestion that Ume6 recruits Ime1 to early meiosis-specific promoters, thus allowing their activation (7, 9, 22, 24). We investigated the association of Ime1 with Ume6 at early gene promoters using ChIP. Ume6-MYC protein-DNA complexes were immunoprecipitated and then reprecipitated using an anti-HA antibody recognizing Ime1-HA. Cells actively proliferating in rich growth medium (YEPD medium) displayed a very low signal for Ime1-HA at either of the early gene promoters *IME2* and *SPO13*, consistent with the lack of expression of Ime1 in growing cells (Fig. 5A). In contrast, cells growing in acetate medium (YEP medium supplemented with 1% potassium acetate [YPKAc]) displayed an Ime1-HA signal at the *IME2* and *SPO13* promoters with no detectable signal for the control *TEL6* sequence. The occupancy of Ime1-HA at the *IME2* and *SPO13* promoters increased following induction of the sporulation program, reaching an approximately 50-times increase at the *IME2* URS1 sequence after 3.5 h, at the time that early gene expression is induced (Fig. 5A and B). These data indicate that Ime1 and Ume6 are both resident on early meiosis-specific gene promoters at the time these genes are induced.

During vegetative growth of S. cerevisiae, the histone deacetylase Rpd3 and the corepressor Sin3, which are tethered to Ume6, impose repression on the early meiosis-specific gene family. Upon entry into the sporulation program, the early genes are activated, and we anticipated that the arrival of Ime1 might trigger the displacement of Rpd3 to allow activation of the meiosis-specific genes. As anticipated, sequential ChIP (ChIP re-ChIP) experiments with anti-MYC antibodies recognizing Ume6-MYC followed by anti-HA antibodies recognizing Rpd3-HA revealed that Rpd3-Ume6 complexes are present at the *IME2* and *SPO13* promoters in cultures proliferating in YEPD or YPKAc medium (Fig. 5C). Surprisingly, the *IME2* and *SPO13* PCR signals from the Rpd3-Ume6 complexes were not reduced as the cultures were induced to initiate the sporulation program. Rpd3-Ume6 complexes remained associated with the *IME2* and *SPO13* promoters after 1 and 3.5 h following induction of sporulation, when *IME2*, *SPO13*, and other early meiosis-specific genes are strongly induced (Fig. 5C and D). ChIP performed with anti-Rpd3 antibodies has yielded similar results (47). One explanation for this observation is that upon induction of the sporulation program, Ime1 binds to Ume6-Rpd3 complexes and is able to activate the target genes. Alternatively, it could be that in a portion of the cells, Ume6 retains Rpd3 and holds the early meiosis-specific genes in a repressed state, while Ime1 binds Ume6 to activate *IME2*, *SPO13*, and the other early genes in a distinct subset of the population. The latter explanation seems unlikely in this strain background, based on the high frequency and synchrony with which cells complete the sporulation process. These observations suggest not only that Ime1 binds to Ume6 coincident with the activation of early meiosis-specific genes but that complexes containing Ume6 and both Rpd3 and Ime1 can form on the early gene promoters.

### Ime1 is required for Gcn5 occupancy of an early meiosis-specific promoter.

Gcn5 is required for induction of early meiosis-specific genes, and cells lacking functional Gcn5 display a defect in progression into the sporulation program (40). Early meiosis-specific-gene induction is also dependent upon Ime1. To test the hypothesis that Gcn5 might be required for Ime1 to activate the reporter gene, LexA-Ime1 was expressed in either a *GCN5* or a *gcn5*::*natMX4* strain harboring a LexA operator-regulated LacZ reporter gene, and the levels of β-galactosidase activity were compared. The LexA-Ime1 fusion effectively induced the expression of the LacZ reporter gene, whereas LexA alone induced very little β-galactosidase activity (Table 1). Deletion of *GCN5* reduced the ability of LexA-Ime1 to activate the reporter, as shown by the greater than 5-fold reduction in β-galactosidase activity detected (741 ± 165.6 [mean ± standard deviation] versus 137 ± 9.2; *P = *0.0029) (Table 1). Deletion of *GCN5* significantly reduced the expression of the LacZ reporter gene, suggesting that Ime1 may be required for Gcn5 to act at early meiosis-specific gene promoters to induce their activation.

**TABLE 1:** β-Galactosidase activity driven by LexA-Ime1 fusion^*a*^

Strain description	Mean β-galactosidase activity ± SD (Miller units)	
	LexA	LexA-Ime1
*GCN5*	6.9 ± 2.7	741 ± 165.7
*gcn5*::*natMX4*	5.85 ± 1.1	137 ± 9.2

a The reporter gene (*8×LexAop*-LacZ) was carried on high-copy-number plasmid pSH18-34. Six independent transformants were analyzed for each condition.

**FIG 6:**
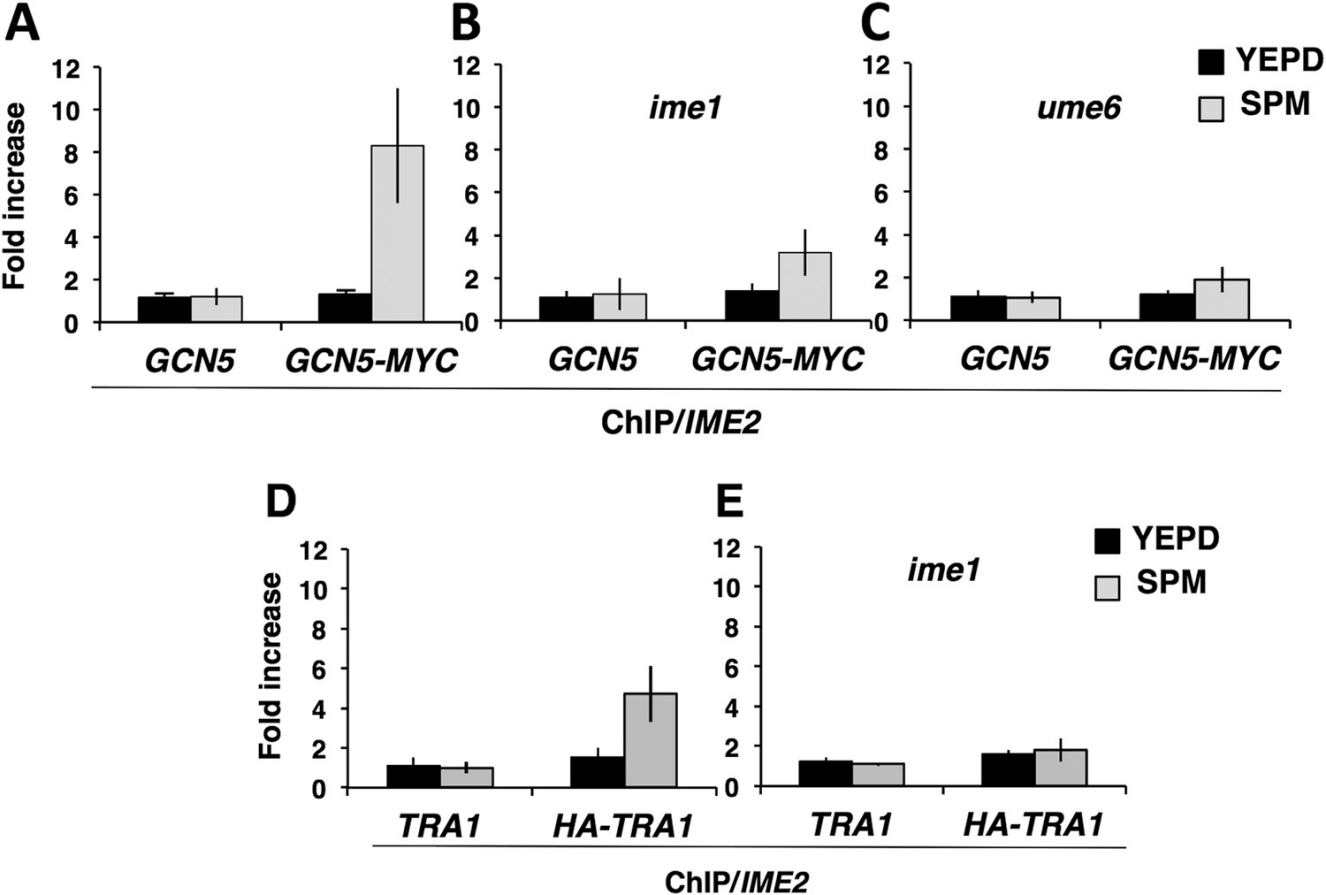
Gcn5 and Tra1 are present at the *IME2* promoter during sporulation. Strains expressing untagged *GCN5* or *GCN5-MYC* (A to C) or untagged *TRA1* or *HA-TRA1* (D to E) during vegetative growth (YEPD) or 3 h post-induction of sporulation (SPM) were subjected to ChIP using anti-MYC or anti-HA antibodies. The fold increase reflects the increase in the *IME2* URS1 PCR product relative to the negative-control *TEL6* sequence that does not harbor a URS1 sequence. (A, D) Wild type. (B, E) *ime1*/*ime1*. (C) *ume6*/*ume6*. The data presented reflect mean values from three independent ChIP replicates and three technical replicates of each sample; errors bars indicate standard deviations.

To further investigate the possibility that Ime1 was required for Gcn5 to participate in the activation of early meiosis-specific gene promoters, we performed ChIP with MYC-tagged Gcn5 to determine whether Gcn5 was resident at the *IME2* promoter at early times during the sporulation program when *IME2* was actively transcribed. Quantitative PCR (qPCR) analysis of Gcn5-MYC immunoprecipitates revealed little enrichment for *IME2* promoter DNA in cells mitotically proliferating in YEPD rich growth medium when *IME2* is not expressed (Fig. 6A). In contrast, *IME2* promoter DNA was enriched more than 6-fold above its level in the no-antibody control in Gcn5-MYC immunoprecipitates prepared 3 h following the induction of the sporulation program (Fig. 6A). No enrichment of *IME2* promoter DNA could be detected when ChIP was performed with an untagged *GCN5* strain, either during mitotic proliferation or sporulation (Fig. 6A). If Ime1 were required for Gcn5 occupancy of *IME2* promoter DNA during sporulation, we predicted that deletion of *IME1* or *UME6* would reduce the presence of Gcn5 at the *IME2* promoter. This hypothesis was tested by performing ChIP analysis with *GCN5-MYC*/*GCN5-MYC ime1*/*ime1* diploids that had been induced to initiate the sporulation program. While *IME2* promoter DNA was readily detected in anti-MYC antibody immunoprecipitates from a *GCN5-MYC*/*GCN5-MYC IME1*/*IME1* diploid (Fig. 6A), the enrichment in the *ime1*/*ime1* diploid was reduced to 2-fold above the level in the no-antibody control (Fig. 6B). No enrichment of *IME2* promoter DNA could be detected when the experiment was performed with strains expressing untagged *GCN5*. Similarly, ChIP analysis demonstrated reduced Gcn5 occupancy of the *IME2* promoter in a *ume6*/*ume6* strain 3 h after induction of the sporulation program (Fig. 6C). The reduction in binding of Gcn5-MYC to the *IME2* promoter appears to be greater in the *ume6*/*ume6* strain than in the *ime1*/*ime1* strain, implying that Ume6 or some component of the chromatin-bound Ume6 complex may promote binding of Gcn5 to the *IME2* promoter in the absence of Ime1. However, the degree of variance among replicate experiments makes it difficult to quantitatively support that conclusion. The apparent reduced presence of Gcn5 at the *IME2* promoter in mutants lacking either *IME1* or *UME6* is consistent with DNA-bound Ume6 acting as a platform that is required for both Ime1 and Gcn5 to occupy the *IME2* promoter.

### Tra1 binds to early meiosis-specific gene promoters and may link SAGA to the Ime1 transcriptional activator.

**FIG 7:**
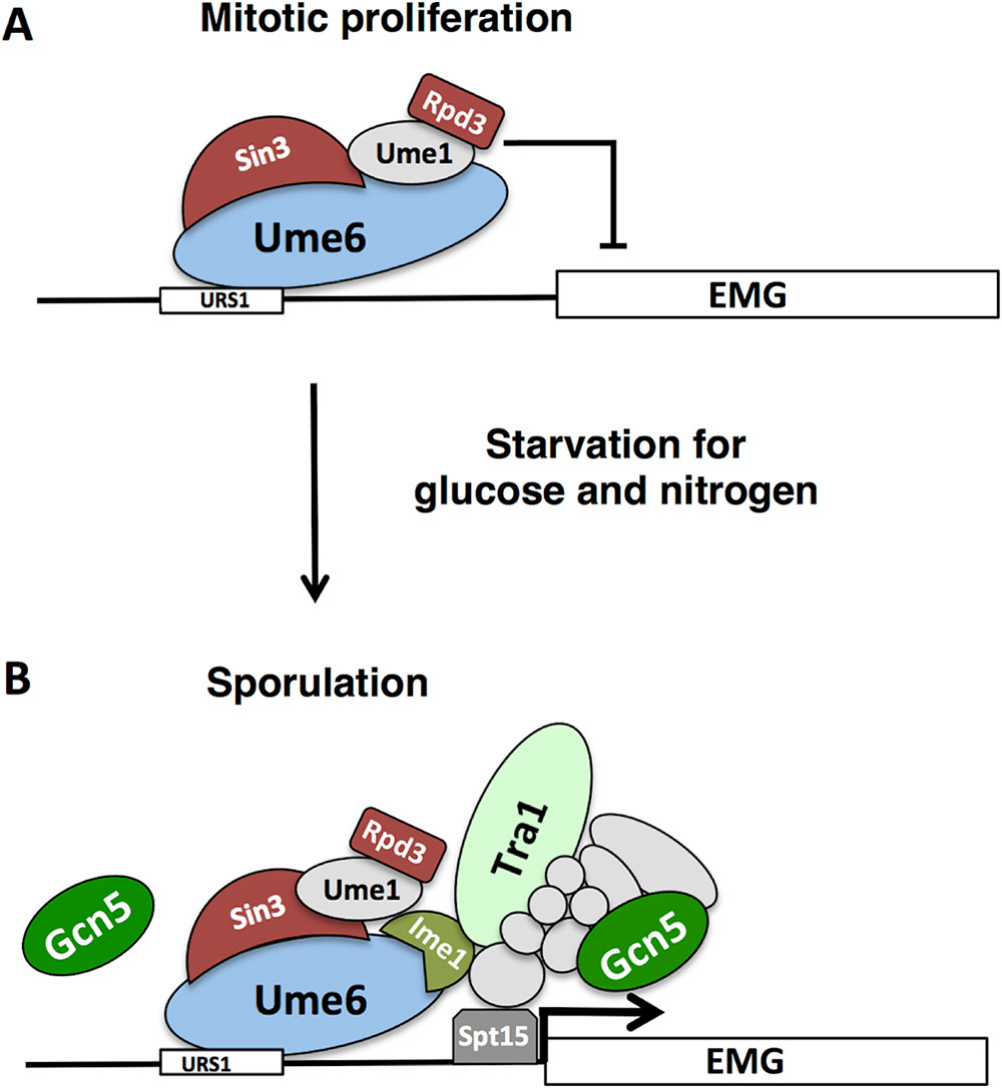
Regulation of early meiosis-specific genes. (A) During mitotic proliferation, early meiosis-specific genes (EMG) are repressed through the action of Ume6 binding to the URS1 DNA sequence. Ume6 acts as a platform to tether repressors Sin3, Rpd3 and other accessory proteins to targeted promoters. (B) Upon starvation for glucose and nitrogen, Ime1 is expressed and binds to Ume6. Our data suggest that Ume6 remains bound to the URS1 sequences of early meiosis-specific genes *IME2* and *SPO13* and that Rpd3 remains associated with Ume6 following Ime1 binding. Interaction of Ime1 with Ume6 is essential for the induction of EMGs in a Gcn5-dependent fashion. Tra1 binds the *IME2* promoter in an Ime1-dependent fashion, raising the possibility that Ime1 directly binds to Tra1, possibly within the context of the SAGA complex. Gcn5 is essential for the induction of meiosis-specific genes and may function in conjunction with Tra1 as a part of the SAGA complex that aids in loading the TATA box binding protein Spt15. Gcn5 may also act directly on Ume6, but it is unclear whether it does so as a part of SAGA or through a distinct protein complex.

Gcn5 is a component of the 19-subunit SAGA complex that promotes gene activation (48). The Tra1 subunit of this complex directly binds to transcriptional activators Gal4 and Gcn4 and thus brings Gcn5 and other SAGA subunits to the cognate promoters (49, 50). We reasoned that Gcn5 might occupy the *IME2* promoter as a component of the SAGA complex and tested the possibility that Tra1 bound to the Ume6-Ime1 complex on the *IME2* promoter. ChIP analysis was performed with an *HA-TRA1*/*HA-TRA1* strain. Anti-HA antibody immunoprecipitates from an untagged *TRA1*/*TRA1* strain revealed no enrichment for the *IME2* promoter DNA in either mitotically proliferating cells or sporulating cells (Fig. 6D). In contrast, anti-HA immunoprecipitates from an *HA-TRA1*/*HA-TRA1* strain displayed a significant increase in the presence of *IME2* promoter DNA in extracts made from sporulating cells but not in extracts from mitotically proliferating cells (Fig. 6D). To further test the hypothesis that DNA-bound Ume6 binds Ime1 and that Ime1 is required for Tra1 binding to the *IME2* promoter, ChIP was performed on extracts from an *HA-TRA1*/*HA-TRA1 ime1*/*ime1* strain or an untagged *TRA1*/*TRA1 ime1*/*ime1* strain 3 h after induction of the sporulation program. No enrichment for *IME2* promoter DNA was detected in immunoprecipitates from extracts of proliferating cultures (Fig. 6D and E). Similarly, there was no enrichment of *IME2* promoter DNA associated with the *HA-TRA1* immunoprecipitated from an *ime1*/*ime1* strain compared to the amount from the no-antibody control (Fig. 6E). Collectively these data support a model whereby DNA-bound Ume6 binds to meiosis-specific activator Ime1, and this in turn is required for Tra1 and SAGA to occupy the *IME2* promoter and, possibly, the promoters of other early meiosis-specific genes (Fig. 7).

## DISCUSSION

### Ume6 remains associated with early meiosis-specific gene promoters during the early phase of sporulation.

The data presented here support a model whereby Ume6 provides a DNA binding platform that directs both positively and negatively acting factors to regulate the expression of early meiosis-specific genes (22, 24). Binding of Sin3 and Rpd3 provides a repressive complex that regulates the chromatin modification state and, possibly, the acetylation state of Ume6 and other proteins in the complex (14, 41). Starvation for nitrogen and glucose induces the expression of Ime1. Modification of Ime1 and Ume6 by Rim11 kinase then promotes association between Ume6 and Ime1, allowing Ime1 to activate the meiosis-specific target genes. Our data support a model whereby this activation is accomplished at least in part through recruitment of Tra1 and the SAGA complex. Based on Western blot analysis from samples prepared under denaturing conditions and on ChIP data, we conclude that Ume6 remains associated with early gene promoters through the early phases of sporulation when early meiosis-specific genes are induced. The repressive function of Ume6 is well documented, and deletion of Ume6 allows derepression of meiosis-specific genes during vegetative growth (17). In contrast, cells lacking Ume6 cannot further induce the expression of early meiosis-specific genes upon starvation and cannot progress effectively into the sporulation program, supporting the contention that Ume6 is required for effective induction of the genes it regulates. Consistent with this is the observation that mutation of URS1, the Ume6 binding site, prevents induction of meiosis-specific genes during sporulation (17, 22). Furthermore, Ime1 is strictly required for induction of the early gene family, and Ime1 binds to Ume6 (17). Indeed, Ime1’s recruitment to early gene promoters and induction of those genes is dependent upon Ume6 (23). The addition of a Gal4 transcriptional activation domain to Ume6 (GAD-Ume6) does not overcome repression of early meiosis-specific genes in vegetative cells, but in cells lacking the repressor Sin3 or Rpd3, GAD-Ume6 can activate a URS1-regulated reporter gene (51). The GAD-Ume6 fusion can rescue the sporulation defect in Ime1-deficient diploids, although not to 100% (24, 51). Further supporting a positive role for Ume6 in meiosis-specific-gene activation is the observation that LexA-Ume6 fusions can activate LexA operator-regulated reporter genes, but only if Ime1 is expressed (22). These observations, in addition to the data reported here, are not consistent with a model where destruction of Ume6 is required to allow activation of the regulated genes. Rather they support a model where Ume6 acts as a binding platform for both repressive factors and the activator Ime1.

It is not entirely clear why our findings differ from previously published observations that Ume6 is rapidly degraded during the early phase of sporulation. In agreement with previous investigations, we observe that Ume6 is more abundant in cells proliferating in rich glucose medium than in cells in acetate medium, but Ume6 is present throughout the early phase of sporulation. Variables that may have yielded some of these differences include the use of different S. cerevisiae strain backgrounds. We made use of SK1 strains, whereas Mallory et al. performed their investigation with an SK1/W303 hybrid (25). Additionally, we made use of different epitope tags and performed protein extractions using a different method. We observe that in vegetative cells, Ume6 is readily detectable by any of the extraction methods performed, but upon initiating sporulation, Ume6 is more difficult to reliably detect with native extraction procedures or by simply boiling cell pellets in SDS sample buffer. It is unclear whether this reflects decreased stability or solubility in the extracts. It may be relevant that in the original description of the method for protein extraction by boiling in SDS sample buffer, the authors noted that protein extraction was much less efficient from cells grown in minimal medium than from cultures grown in rich medium (52).

### APC^Cdc20^ activity is not required for induction of early meiosis-specific genes.

We find no evidence to support a role for APC subunit Cdc20 in the degradation of Ume6 during the early phase of sporulation or induction of the early meiosis-specific genes. Cdc20 accumulates in the middle phase of sporulation rather than at early times, and it serves an essential role in the completion of metaphase I (26, 27). The role of Cdc20 at metaphase I is likely dependent on the proteosome, since inhibition with MG132 yields a similar metaphase I arrest without impeding progression through the early phases of sporulation (37). We observed that meiosis-specific depletion of Cdc20 yielded the expected metaphase I arrest but did not alter the induction of the early meiosis-specific genes. While it is possible that sufficient Cdc20 remained in the cells to execute some function related to gene expression, we did not observe any effects on Ume6.

In contrast, autophagy is required for the early stages of sporulation, and Ume6 is a regulator of *ATG8*, an essential component of the autophagy system (36). While there is no evidence that autophagy leads to destruction of Ume6, the precise role of autophagy in S. cerevisiae sporulation has not been determined. It may be that the process is required to provide precursors for nascent protein synthesis, since the sporulation process occurs in the absence of nitrogen and amino acids.

Although we did not see a dramatic reduction in the Ume6 abundance in the early phase of sporulation (0 to 4 h), we did observe reduction of Ume6 at late times in sporulation where Cdc20 may be more active; however, we have not investigated whether this is dependent on Cdc20 or the putative destruction box sequences in Ume6. This study employed a Cdc20 depletion technique, whereas a previous investigation made use of a thermosensitive *cdc20-1* allele, which increases the complexity of the experiment since some aspects of sporulation are temperature sensitive in most S. cerevisiae strains (25, 53). The difference in methods applied to inactivate Cdc20 may be responsible for some of the differences from our results.

It is entirely possible that Ume6 is targeted for degradation under some conditions or at some gene promoters since it regulates genes that are required for functions other than sporulation (54). Thus, destruction of Ume6 within some contexts may be important for gene regulation. For example, in Candida albicans, Ume6 is an important activator of genes required for hypha formation and undergoes Skp-cullin-F box (SCF)-dependent degradation in response to oxygen or other conditions that inhibit hyphal growth (55, 56). SCF is required for sporulation in S. cerevisiae, but early gene expression is not affected by Cdc53 depletion or Cdc4 inactivation (57).

### Repressor Rpd3 remains associated with Ume6 when early genes are activated.

It was expected that the lysine deacetylase Rpd3 would be associated with Ume6 at early meiosis-specific gene promoters during vegetative growth, but it was surprising to find that it remained associated with these promoters while those genes were induced. We did not probe for the presence of Sin3 in these complexes, but Sin3’s abundance does not change during sporulation and Ume6-Sin3 complexes have been detected during early and middle phases of sporulation (47, 58). Sin3 physically interacts with Ume6 through contact with specific residues in a Sin3 Pah domain (51). In contrast, there is no evidence for direct binding of Rpd3 to Ume6. Rpd3 is resident in multiple chromatin-bound complexes in cells (46). Indeed, interaction between Rpd3, Sin3, and other subunits of the Ume6 complex may be critical to the recruitment and retention of Rpd3 on early meiosis-specific gene promoters (15, 59). Distinct from the findings of Inai et al. (47) and those reported here, Pnueli et al. observed transient displacement of Rpd3 from the *IME2* promoter at the onset of sporulation (23). Those investigators monitored the Rpd3-13×MYC occupancy of *IME2* carried on a 2μ plasmid, which may have given a different result from our monitoring of occupancy of the endogenous *IME2* and *SPO13* promoters by an endogenously tagged Rpd3-3×HA.

We were able to identify Ume6-Rpd3 and Ume6-Ime1 complexes bound to early meiosis-specific gene promoters during the early phase of sporulation, and there is evidence that Ime1 and Rpd3 co-reside in at least a subset of those complexes. A simple interpretation of these data would suggest that Rpd3 remains bound to Ume6 following the recruitment of Ime1. However, the possibility that Rpd3 is transiently displaced but not easily detectable within the cell population at the level of resolution allowed by our synchronization protocol cannot be precluded. A caveat to this aspect of our investigation is that Rpd3 binds to several complexes, not only Ume6, and has been shown to spread over some chromosomal regions (46). It is possible that Rpd3 does dissociate from Ume6 but remains in close proximity, associated with other chromatin complexes, and our procedure does not have sufficient resolution to distinguish between them.

It is unclear exactly how the repressive effects of Sin3-Rpd3 are bypassed when Ime1 joins the Ume6 complex during the sporulation process. It may be that binding to Ime1 leads to rearrangement of the three-dimensional structure of the complex, rendering Rpd3 unable to deacetylate surrounding chromatin or relevant proteins within the complex. Alternatively, Ime1 or some other meiosis-specific factor might inhibit Rpd3 activity. Rpd3L complexes containing Ume6 and Rpd3 display reduced histone deacetylase activity when cells are cultured in acetate medium (60). The repressive activity of Rpd3 is dependent on the activator it competes with and the degree of activation (61). Thus, it may simply be that Ime1 is a sufficiently strong activator to overpower the repressors while it is bound to Ume6.

Rpd3-Sin3 in conjunction with Isw2 promotes an inhibitory chromatin structure that masks access to the TATA sequence of meiosis-specific genes *IME2* and *HOP1* (62). This inhibitory chromatin structure is overcome by recruitment of Ime1, which promotes binding of the TATA box binding protein (TBP) encoded by *SPT15* to the TATA sequence of *HOP1* (62). Recruitment of TBP to the *IME2* promoter by Ime1 has not been specifically demonstrated, but TBP binding is promoted by other transcriptional *trans* activators (39). A variety of structural and genetic data support a model whereby Tra1 as a component of the SAGA complex binds directly to *trans* activators, bringing Gcn5 and TBP to specific promoters to induce transcriptional activation (63).

The expression of early meiosis-specific genes is dependent not only on Ime1 but also on Gcn5. The chromatin surrounding early gene promoters displays an increase in acetylated histones at the onset of sporulation that is dependent upon Gcn5, and the early genes are not induced in *gcn5* mutants (40). The relationship between Ime1 and Gcn5 is not entirely clear. We observe that Gcn5’s association with the *IME2* promoter is largely though not entirely dependent upon Ime1, suggesting that Tra1 and the SAGA complex may bind Ime1 as they do with the activators Gal4 and Gcn4 (38, 49). This observation does not preclude the possibility that other transcriptional activating complexes also participate in this process. Gcn5 is known to be a component of the SAGA, SLIK, and ADA complexes (64, 65). This interpretation is subject to several caveats, as we have not demonstrated direct interaction between Tra1 and Ime1 or any component of the Ume6 complex. Furthermore, the Ume6-Isw2 complex can in some cases promote chromatin loop formation, which could potentially allow cross-linking with proteins associated with the promoters of genes that are not meiosis-specific and cloud the interpretation of results at specific promoters (66).

Ime1 may not be strictly required for histone acetylation surrounding the *IME2* promoter, as some increase in histone H3 acetylation has been reported in *ime1* mutants that are incubated in sporulation medium (47). In *gcn5* mutants, there is some evidence that Ime1 does not bind stably to promoter chromatin, suggesting that acetylation of histones or some component of the Ume6 complex is required for stable Ime1 interaction (47). It is clear that Gcn5 is required for more than reversal of Rpd3-dependent histone deacetylation, since Gcn5 is required for sporulation even in the absence of Rpd3 (40). In addition to histones, Gcn5 can acetylate Ume6 (41), and it is possible that acetylation of Ume6 is required for Ime1 to associate stably with the complex. This scenario might explain the failure to induce early genes in a *gcn5* mutant and the weak induction of early genes in a strain harboring a mutated Ume6 that cannot be acetylated (40, 41).

The data reported here support a role for Ume6 as a binding platform for both activators and repressors of early meiosis-specific genes. Stable association of Ume6 with chromatin throughout the sporulation process is important for the activation of early genes and may be important for the ability to reimpose repression as cells progress into the middle and late phases of sporulation. Additionally, Ume6 plays an important role in spore germination (67). Thus, retaining the complex on chromatin and regulating meiosis-specific expression through the stability and posttranslational regulation of the activator Ime1 may be more economical for cells than dissociating or degrading the DNA binding complex, since this would require resynthesis of Ume6 and reassembly of the repressive complex during the late phase of sporulation when cells do not have access to external sources of nitrogen.

## MATERIALS AND METHODS

### Strains and plasmids.

The Saccharomyces cerevisiae strains used in this study and their relevant genotypes are listed in Table 2. Strain EGY48 was provided by Erica Golemis, and its construction has been previously described (68). Strain DSY1725 was derived from EGY48 by transformation. Other strains used throughout this work were derived from the SK1 genetic background (69). Unless otherwise indicated, strains in the SK1 background were derived from DSY1030 *MAT***a** *ho*::*LYS2 lys2 ura3 leu2*::*hisG trp1*::*hisG arg4-bgl his4B* and DSY1031 *MAT*α *ho*::*LYS2 lys2 ura3 leu2*::*hisG trp1*::*hisG arg4-nsp his4X*. The SK1 diploid strain A5128, generously provided by Angelika Amon, has the endogenous *CDC20* promoter replaced by the mitosis-specific *CLB2* promoter and includes 3 copies of the HA epitope at the amino terminus of *CDC20* as described previously (26). S. cerevisiae strains were routinely maintained on YEPD rich medium (70). Sporulation experiments were performed as previously described (71). Single colonies of each diploid strain were selected from a YEP-glycerol agar plate to ensure mitochondrial function prior to overnight propagation in YEPD medium. Cultures were diluted 1:100 into YEP medium supplemented with 1% potassium acetate (YPKAc) for overnight growth. Cultures were then collected by centrifugation, washed with water, and resuspended in sporulation medium (SPM) (1% KAc, 0.02% raffinose) to initiate sporulation.

**TABLE 2:** Saccharomyces cerevisiae strains used in this study

Strain	Genotype	Reference or source
DSY1030	*MAT***a** *ho*::*LYS2 lys2 ura3 leu2*::*hisG trp1*::*hisG arg4-bgl his4B*	71
DSY1031	*MATα ho*::*LYS2 lys2 ura3 leu2*::*hisG trp1*::*hisG arg4-nsp his4X*	71
DSY1089	*MAT***a**/α DSY1030 × DSY1031	71
DSY1718	*MAT***a**/α *ime1*::*kanMX4*/*ime1*::*kanMX4*	This study
DSY1721	*MAT***a**/α *ume6*::*kanMX4*/*ume6*::*kanMX4*	This study
DSY1700	*MAT***a**/α *UME6-13×MYC-TRP1*/*UME6-13×MYC-TRP1*	This study
DSY1701	*MAT***a**/α *3×HA-UME6*/*3×HA-UME6*	This study
DSY1705	*MAT***a**/α *UME6-13×MYC-TRP1*/*UME6-13×MYC-TRP1 RPD3-3×HA-kanMX4*/*RPD3-3×HA-kanMX4*	This study
DSY1707	*MAT***a**/α *UME6-13×MYC-TRP1*/*UME6-13×MYC-TRP1 IME1-3×HA-kanMX4*/*IME1-3×HA-kanMX4*	This study
DSY1732	*MAT***a**/α *GCN5-13×MYC-TRP1*/*GCN5-13×MYC-TRP1 ime1*::*kanMX4*/*ime1*::*kanMX4*	This study
DSY1735	*MAT***a**/α *GCN5-13×MYC-TRP1*/*GCN5-13×MYC-TRP1 ume6*::*kanMX4*/*ume6*::*kanMX4*	This study
DSY1421	*MAT***a**/α *GCN5-13×MYC-TRP1*/*GCN5-13×MYC-TRP1*	This study
DSY1831	*MAT***a**/α *SIN3-13×MYC-TRP1*/*SIN3-13×MYC-TRP1*	This study
DSY1841	*MAT***a**/α *TRP1-3×HA-TRA1*/*TRP1-3×HA-TRA1*	This study
DSY1845	*MAT***a**/α *TRP1-3×HA-TRA1*/*TRP1-3×HA-TRA1 ime1*::*kanMX4*/*ime1*::*kanMX4*	This study
A5128	*MAT***a**/α P*CLB2-3×HA-CDC20*/P*CLB2-3×HA-CDC20*	26
EGY48	*MAT*α *his3 trp1 ura3-52 leu2*::*6×LexAop*::*LEU2*	80
DSY1725	EGY48 *gcn5*::*natMX4*	This study

The *HA-UME6* allele, generously provided by Aaron Mitchell, has 3 tandem copies of the HA epitope introduced into the Ume6 sequence at amino acid 122 (43). The tagged construct was used to replace the endogenous *UME6* gene through a two-step gene replacement procedure (72). The carboxyl-terminal MYC or HA tags were introduced into the endogenous *UME6*, *RPD3*, *SIN3*, *IME1*, and *GCN5* open reading frames by PCR-mediated transformation (73). A 900-bp fragment of the *TRA1* promoter was amplified from SK1 genomic DNA with oligonucleotides Tra1p5/Tra1p3 and inserted into BglII-/PacI-cut pFA6a-*TRP1-GAL1*-3xHA. This was used as a template for amplification with Tra1Up2/Tra1dwn. The PCR product was used to tag *TRA1* (72). Deletion of the *UME6*, *IME1*, and *GCN5* genes was accomplished by PCR-mediated transformation (74). Oligonucleotide DNA sequences used to generate tagged genes and gene deletions are listed in Table 3. The modified strains, with the exception of DSY1725 (EGY48 *gcn5*::*natMX4*), were subjected to two rounds of backcrossing to the parent DSY1030 to ensure that no unwanted mutations had been acquired during the strain construction.

**TABLE 3:** Oligonucleotides used in this study

Name	Sequence	Gene
SPO13p5	TTCAAGCTTCTTGATTTACC	*SPO13*
SPO13p3	ACCCTAATTCGAGTAGCCTA	*SPO13*
IME2p5	CTCTCACAAAAATCTGAGTG	*IME2*
IME2p3	GACAGAAGAAGTTATTCAGG	*IME2*
IME2SC5	TTACAGTTACCTTTACTTTACC	*IME2*
IME2SC3	ATAAATGACCTATTAAGTTAAGC	*IME2*
HOP1p5	AAGTGGAGTTAACGTTGTGG	*HOP1*
HOP1p3	TGTATATGTCTTGTAAATCA	*Hop1*
TEL6A	CTCGTTAGGATCACGTTCGA	*TELVI*
TEL6B	ACGACTTCGTCTCAGAAGAG	*TELVI*
TEL6C	CATTGTGGCTTTGTTACGC	*TELVI*

Epitope-tagging primers		
IME1T3	ATATATGCAAATACACAAATTCTTAGCAATTTGAGTGAGACAATGGAAATAAAGAAATGAGAATTCGAGCTCGTTTAAAC	*IME1*
IME1T5	GATTATTATGACAAGGTCAGGTTTCAAGAAATATCCTACAAGTTTAGTAAAACCTATTCTCGGATCCCCGGGTTAATTAA	*IME1*
RPD3T3	AAATTATATTGGCACCGCTTTATCAACAGCGGTGGGACGAGACGTTTAGATAGTAATTACGAATTCGAGCTCGTTTAAAC	*RPD3*
RPD3T5	ACGAAGGGTGGTTCGCAATATGCGAGGGACCTACATGTTGAGCATGACAATGAATTCTATCGGATCCCCGGGTTAATTAA	*RPD3*
UME6T3	TTTCCTTTTGACGCCCAAAGCAACACGGACAGCATGAGGGGAAACAGTCGGAATAATATGGAATTCGAGCTCGTTTAAAC	*UME6*
UME6T5	AAACTGGAGGAAATCAAGAAAAAAACAAAAGAGGCCAAAAGAAGAGCAATGAAAAAAAAACGGATCCCCGGGTTAATTAA	*UME6*
SIN3T3	TACAATTTTAAAATTACAATGTTATATCGTTGACATTAATTAAAGGTACACATCAGAAGAGAATTCGAGCTCGTTTAAAC	*SIN3*
SIN3T5	GATGATAATATAGAAACGACTGGGAATACTGAATCTTCAGACAAGGGGGCTAAGATTCAACGGATCCCCGGGTTAATTAA	*SIN3*
GCN5T3	AAAAGTAGTAAAATAACCTCAATTGATCACATCGTCTCGCCGTACTAAACATTTATTTCTGAATCCGAGCTCGTTTAAAC	*GCN5*
GCN5T5	CTAGAGAAATTCTTCAATAATAAAGTAAAAGAAATACCTGAATATTCTCACCTTATTGATCGGATCCCCGGGTTAATTAA	*GCN5*
Tra1p5	CGACACGCGGATCCGTAGATTCATCAAGAGAGAGC	*TRA1*
Tra1p3	GCGAGATTCGGTTAATTAAAGACATCGGCAAAATGCGG	*TRA1*
Tra1Up2	GTGTAGTGTAGGGTGAATTGCCCAAAGTAGGAACAGTGTCCGAGGAAAAGTTATGATATGCCTCCTTACGCATCTGTGC	*TRA1*
Tra1dwn	GAGTGGCATCATCATCGCGAAACCTACTGGCGAATTGCTCGATCTGCTCAGTGAGTGATCCAGCGTAATCTGGAACGTC	*TRA1*

Gene deletion oligonucleotides		
UME6d5	CAGCGCACAGGAACTAGGACACTACCGCACTCAAACCATTTGCATGGACCTTAACTCACGAAGCTTCGTACGCTGCAGG	*UME6*
UME6d3	AATGACAGTAATAATAATAATAATAGTAACAATATCTCTTTTTTTTTTTCAGTGAGCTTTCGACTCACTATAGGGAGACC	*UME6*
IME1d5	GGTGAAAAAGGAAAAAAATAATAAAAGAAAAGCTTTTCTATTCCTCTCCCCACAAACAAAAAGCTTCGTACGCTGCAGG	*IME1*
IME1d3	AAATGAGTGTGAATGGATATATTTTGAGGGAAGGGGGAAGATTGTAGTACTTTTCGAGAACGACTCACTATAGGGAGACC	*IME1*
GCN5d5	AAGACCGTGAGCCGCCCAAAAAGTCTTCAGTTAACTCAGGTTCGTATTCTACATTAGATGAAGCTTCGTACGCTGCAGG	*GCN5*
GCN5d3	CGTACTAAACATTTATTTCTTCTTCGAAAGGAATAGTAGCGGAAAAGCTTCTTCTACGCACGACTCACTATAGGGAGACC	*GCN5*

### Cytology.

Sporulation frequency was determined by counting asci visualized by light microscopy; 200 cells per culture were counted. Progression through sporulation was monitored by fluorescence microscopy. At each time point, 100 μl of culture was fixed in 70% ethanol for at least 4 h at 4°C. Following rehydration, cells were stained with 0.5 μg/ml 4′,6-diamidino-2-phenylindole (DAPI) and visualized; 200 cells per time point were counted. Cells that contained two or more masses of chromatin stained with DAPI were considered to be post-MI.

### Protein analysis.

Samples of culture were collected at the indicated time points and protein extracts prepared by bead beating in 20% trichloroacetic acid (TCA) as described previously (75). The cells were pelleted by centrifugation for 1 min at 10,000 × *g*. The medium was removed, and cell pellets were resuspended in 500 μl of 20% TCA and incubated for 2 min on ice. Cell samples were pelleted by centrifugation, the TCA removed, and the cell pellet resuspended in 200 μl of 20% TCA. Glass beads (0.5 mm) were added to each sample, and the cells were disrupted by vortexing 4 times for 1 min each at full speed and incubating for 1 min on ice between bursts of vortexing. Four hundred microliters of 5% TCA was then added to each sample for a final mixture of 10% TCA. The bottom of each tube was then punctured with a 25-gauge needle and each tube placed into a fresh tube before centrifuging for 3 min at 3,000 × *g* to separate the cell extract from the beads. The cell extracts were then subjected to centrifugation for 10 min at 14,000 × *g*. The TCA was removed, and the pelleted extract was resuspended in 1 ml of ice-cold acetone. This was again subjected to centrifugation for 10 min at 14,000 × *g*. The acetone was removed, and the pellet resuspended in 100 μl of 4% SDS, 20% glycerol, 0.02% bromophenol blue, 100 mM Tris-HCl (pH 6.8), 20 μl of 1 M dithiothreitol (DTT), 30 μl of 1 M Tris base, 50 μl H_2_O. Samples were stored at −80°C prior to analysis by gel electrophoresis. An alternative protein extraction procedure was performed as described previously (45). The cell pellets taken at each time point were resuspended in 1 ml of ice-cold water, and then 150 μl of 1.85 N NaOH, 7.5% 2-mercaptoethanol was added. This mixture was incubated on ice for 10 min prior to the addition of 150 μl of 55% TCA. Following a 10-min incubation on ice, the extract was centrifuged for 10 min at 14,000 × *g*. The supernatant was removed, and the pellet was resuspended in 100 μl HU buffer (8 M urea, 5% SDS, 200 mM Tris-HCl [pH 6.8], 1 mM EDTA, 0.1% bromophenol blue, DTT added to a final concentration of 1.5% immediately before use)/2 × 10^7^ cells. Protein extracts were prepared under nondenaturing conditions by resuspending cell pellets in 50 mM Tris-HCl (pH 7.4), 300 mM NaCl, 0.5% NP-40, 10% glycerol (pH 8.0), 20 μg/ml each of pepstatin, leupeptin, and aprotinin, 0.5 mM phenylmethylsulfonyl fluoride (PMSF), 10 mM sodium fluoride, 60 mM β-glycerophosphate, 10 mM sodium pyrophosphate, 5 mM EDTA, 5 mM EGTA. Glass beads (0.5 mm) were added to each sample, and the cells were disrupted by vortexing 4 times for 1 min each time at full speed and incubating for 1 min on ice between bursts of vortexing. The extracts were subjected to centrifugation for 10 min at 14,000 × *g*, and the soluble portion was mixed with an equal volume of 4% SDS, 20% glycerol, 200 mM DTT, 0.02% bromophenol blue, 100 mM Tris-HCl, pH 6.8.

Ume6 protein samples were separated by gel electrophoresis using 15-cm 8% SDS–polyacrylamide gels. Proteins were transferred to polyvinylidene fluoride (PVDF) membranes (ImmobilonP) using semidry transfer (48 mM Tris, 39 mM glycine 0.037% SDS, 20% methanol) for 90 min at 200 mA. The membranes were subsequently cut at the 55-kDa marker and separated. The upper half of each membrane was probed for Ume6 by incubating with antibody recognizing the HA or MYC epitope tag or Ume6 as indicated. The lower half of each membrane was probed for Cdc28 by incubation with an anti-PSTAIRE antibody recognizing the amino acid sequence EGVPSTAIREISLLKE in Cdc28. The antibodies used were as follows: anti-HA mouse monoclonal antibody HA.11 (clone 16B12, MMS-101R; Covance), 3 mg/ml ascites fluid used at 1:10,000 dilution; anti-MYC mouse monoclonal antibody 9E10 (MMS-150R; Covance), 4.1 mg/ml ascites fluid used at 1:10,000 dilution; affinity-purified anti-Ume6 chicken polyclonal antibody (GW22454A, batch number 039K2073V; Sigma-Aldrich), 1 mg/ml used at 1:500 dilution; and anti-PSTAIRE mouse IgG1 monoclonal antibody (P7962, lot number 010M4766; Sigma-Aldrich), 7.2 mg/ml ascites fluid used at 1:10,000 dilution. Primary anti-HA and anti-MYC antibodies were detected with horseradish peroxidase (HRP)-conjugated goat anti-mouse IgG (12-349; Millipore Sigma). Primary anti-Ume6 antibody was detected with HRP-conjugated anti-chicken IgY (A9046, batch number 113M4824). For the quantitative Western blot analysis whose results are shown in Fig. 1, the same primary antibodies were employed; these were detected with the following IRDye-coupled secondary antibodies as appropriate: IRDye 680RD donkey anti-chicken IgY 926-68075 (Ume6) or IRDye 680RD goat anti-mouse IgG 926-68070 (Ume6-MYC) (LI-COR Biosciences). The Odyssey FC infrared imaging system scanner (LI-COR Biosciences) was used to visualize the decorated proteins in the 700-nm and 800-nm channels. Quantification of the band intensities was performed using ImageJ, and the relative amount of Ume6 or Ume6-MYC was normalized to the amount of the loading control Cdc28. Sin3-MYC samples were separated in 6.4-cm 6% SDS–polyacrylamide gels. Rpd3-HA samples were separated in 6.4-cm 10% SDS–polyacrylamide gels. Western blot detection was performed using the appropriate primary antibody and HRP-conjugated secondary antibodies as described above. Duplicate samples were electrophoresed in 10% SDS–polyacrylamide gels for Western blot detection of Cdc28.

### Analysis of gene expression.

Samples of total RNA collected from actively proliferating cells and at intervals following the induction of sporulation were analyzed by Northern blotting using ^32^P-labeled PCR-generated probe fragments as previously described (76). Repression of the P*IME2*-LacZ reporter and activation of the *LEXAop*-LacZ reporter was monitored by *p*-nitrophenyl-α-d-galactopyranoside (ONPG) assay as described previously (77). Six independent cultures of each strain actively proliferating in SD-ura medium (70) were assayed. Statistical significance was determined by the use of an unpaired *t* test using the GraphPad *t* test calculator.

### Chromatin immunoprecipitation.

Chromatin immunoprecipitation (ChIP) was performed based on previously described protocols (78). Samples of 2 × 10^8^ cells, either mitotically proliferating or sporulating, were collected by centrifugation and washed with phosphate-buffered saline (PBS), followed by cross-linking for 45 min by the addition of 10 mM dimethyl adipimidate (DMA) in PBS plus 0.25% dimethyl sulfoxide (DMSO). The DMA was subsequently washed out, and the cells were subjected to further cross-linking in the presence of 1% formaldehyde for 60 min. The cross-linking reaction was quenched by the addition of glycine to a final concentration of 125 mM. The samples were washed four times in 4 volumes of PBS. Cell pellets were lysed by beating with 0.5-mm glass beads in FA lysis buffer (50 mM HEPES [pH 7.5], 150 mM NaCl, 1 mM EDTA, 1% Triton X-100, 0.1% Na-deoxycholate, 0.1% SDS, 20 μg/ml each of aprotinin, leupeptin, pepstatin, 1 mM PMSF), and chromatin was sheared by sonicating 10 times for 15 s each time with a 15 s rest on ice between bursts to achieve an average size of 300 to 400 bp. Following centrifugation for 10 min at 13,000 × *g*, 1/10 of each lysate was reserved as a whole-cell extract (WCE) control and the remaining supernatant fraction was precleared by the addition of protein G-Sepharose beads that had been blocked with salmon sperm DNA. The target proteins were immunoprecipitated with either 100 μg anti-MYC antibodies (9E10, MMS150R; Covance) or 100 μg anti-HA antibodies (HA.11, MMS-101; Covance) in ascites fluid and salmon sperm-blocked protein G-Sepharose beads. The antibody-bead complexes were washed once with FA lysis buffer, once with FA lysis buffer supplemented with NaCl to a final concentration of 500 mM, once with ChIP wash buffer (10 mM Tris-HCl at pH 8.0, 0.25 M LiCl, 1 mM EDTA, 0.5% NP-40, 0.5% [wt/vol] sodium deoxycholate), and twice with 10 mM Tris at pH 8.0, 1 mM EDTA, for 5 min each time. Protein-DNA complexes were eluted using ChIP elution buffer (50 mM Tris-HCl at pH 8.0, 10 mM EDTA, 1% SDS). Cross-linking was reversed by incubation at 95°C for 10 min and 65°C overnight followed by proteinase K digestion at 42°C for 3 h. The WCE samples were prepared similarly. DNA was subsequently isolated using PCR purification spin columns (Qiagen). For ChIP re-ChIP experiments, the initial immunoprecipitate was incubated in ChIP elution buffer for 30 min, followed by centrifugation. The supernatant was diluted to 1 ml with FA buffer and was reprecipitated with the second antibody. The DNA samples recovered were subjected to 15 cycles of PCR with ^32^P-end-labeled oligonucleotide pairs (2 pmol of each oligonucleotide). Oligonucleotide sequences used for PCR analysis are listed in Table 3. The *SPO13* oligonucleotide pair amplifies a DNA fragment, including the URS1 at −96. The *SPO13* pair amplifies a DNA region encompassing URS1 sequences at −456 and −551. The *TELVI* oligonucleotide pair amplifies a fragment from the right arm of the chromosome VI telomere that has no URS1 sequence. The PCR products were separated on 6% polyacrylamide gels, and a Molecular Dynamics STORM 840 phosphor imager was used to scan and quantitate the radioactive signals. To determine relative enrichment, the PCR signals are reported as the ratios of immunoprecipitated DNA/input DNA. All ChIP analyses were performed in triplicate.

Chromatin immunoprecipitation experiments for Gcn5 and Tra1 were analyzed by qPCR. Chromatin immunoprecipitation was performed as described above, but 2% of the cell lysate was reserved as the input control. The remaining extract was split, and half was subjected to immunoprecipitation with anti-MYC or anti-HA antibodies in ascites fluid (10 μg) and protein G-Sepharose while half received no antibodies (no antibody control). The input and immunoprecipitated DNA were subjected to qPCR analysis as follows: total volume of 10 μl containing 10 pmol of forward and reverse primers (IME2SC5/IME2SC3 or TEL6B/TEL6C), 5.0 μl 2× iQ SYBR green supermix (Bio-Rad), and 2 μl of the sample as the template. Amplifications were performed using the Rotor-Gene Q real-time PCR cycler. No-template controls were included in each reaction mixture, and all samples were tested in technical triplicates. The amplification efficiency of all primer pairs was tested prior to use with serial dilutions of sonicated chromosomal DNA to confirm that amplification efficiency was greater than 97%. Enrichment was calculated, as previously described (79), by determining the percentage of input DNA recovered in each *IME2* promoter-specific immunoprecipitation minus no-antibody control divided by input DNA. The values recovered from the *IME2* promoter immunoprecipitations were then divided by the percentages of input DNA recovered from the *TELVI* immunoprecipitations. Data shown reflect three independent immunoprecipitations and three technical replicates of each. Error bars indicate standard deviations.
